# Unusual sequence length-dependent gold nanoparticles aggregation of the ssDNA sticky end and its application for enzyme-free and signal amplified colorimetric DNA detection

**DOI:** 10.1038/srep30878

**Published:** 2016-08-01

**Authors:** Hongfei He, Jianyuan Dai, Zhijuan Duan, Baozhan Zheng, Yan Meng, Yong Guo, Dan Xiao

**Affiliations:** 1 College of Chemistry, Sichuan University, 29 Wangjiang Road, Chengdu 610064, People’s Republic of China; 2 College of Chemical Engineering, Sichuan University, No. 24 South Section 1, Yihuan Road, Chengdu 610065, People’s Republic of China

## Abstract

It is known that the adsorption of short single-stranded DNA (ssDNA) on unmodified gold nanoparticles (AuNPs) is much faster than that for long ssDNA, and thus leads to length-dependent AuNPs aggregation after addition of salt, the color of the solutions sequentially changed from red to blue in accordance with the increase of ssDNA length. However, we found herein that the ssDNA sticky end of hairpin DNA exhibited a completely different adsorption behavior compared to ssDNA, an inverse blue-to-red color variation was observed in the colloid solution with the increase of sticky end length when the length is within a certain range. This unusual sequence length-dependent AuNPs aggregation might be ascribed to the effect of the stem of hairpin DNA. On the basis of this unique phenomenon and catalytic hairpin assembly (CHA) based signal amplification, a novel AuNPs-based colorimetric DNA assay with picomolar sensitivity and specificity was developed. This unusual sequence length-dependent AuNPs aggregation of the ssDNA sticky end introduces a new direction for the AuNPs-based colorimetric assays.

Highly sensitive and selective detection of DNA is increasingly important in clinical diagnostics, forensic investigations, environmental and food safety monitoring^1^. Accordingly, several detection methods have been developed, e.g., fluorescence^2^,^3^, chemiluminescence^4^,^5^, and electrochemistry^6^,^7^. Although these methods can detect exceedingly low levels of DNA, they have limitations with respect to complex operations, tedious labels, high cost, and dedicated instruments. Colorimetric assay is an attractive analytical method due to its simplicity, low cost, easy observation by the naked eye and no requirement of sophisticated instruments^8^. AuNPs have been successfully employed as a colorimetric probe because of their unique localized surface plasmon resonance (SPR) absorption^9^. Most of AuNPs-based assays rely on the covalent modification of AuNPs with thiolated DNA or aptamer and the interparticle cross-linking mechanism^10^. However, the synthesis of thiol-containing ligand-modified AuNPs is an expensive and time-consuming process. Recently, Li and Rothberg reported an interesting phenomenon that single-stranded DNA (ssDNA) and double-stranded DNA (dsDNA) have different propensities to adsorb on unmodified AuNPs^11^. ssDNA can adsorb onto AuNPs via van der Waals attraction between the exposed bases and nanoparticles, and protects AuNPs from salt-induced aggregation, whereas dsDNA cannot do so because of the electrostatic repulsion between their phosphate backbone and the negatively charged AuNPs. This mechanism has been employed for the detection of DNA^11^,^12^,^13^, RNA^14^, protein^15^,^16^, metal ions^17^,^18^ and small molecule^19^. Meanwhile, Li and Rothberg also found that the rate of adsorption onto the AuNPs surface was related to sequence length of ssDNA, short ssDNA adsorb more quickly^20^. Therefore, the adsorption amount of short ssDNA and long ssDNA on AuNPs will be different if ssDNA was incubated with AuNPs in proper short time (e.g., 2 min) and then leads to length-dependent AuNPs aggregation after addition of salt, the color of the AuNPs solutions sequentially changed from red to blue in accordance with the increase of ssDNA length^21^. This observation has been exploited to develop the colorimetric assay for specific sequences and SNPs in PCR-amplified genomic DNA^20^, enzymatic cleavage and oxidative damage of ssDNA without need of covalently modified AuNPs^21^. Although AuNPs-based colorimetric assays have been widely studied due to the advantages mentioned above, they still suffered from the limited sensitivity and poor selectivity. To circumvent this limitation, some enzyme-assisted colorimetric signal amplification approaches have been reported^22^,^23^. However, the use of protein enzymes suffers from many limitations in practical applications because enzymes are expensive, temperature-sensitive and usually sequence-specific. An enzyme-free and signal amplified AuNPs-based colorimetric detection strategy is therefore still highly required. Up to now, most of the employed DNA in AuNPs-based colorimetric assay were ssDNA or dsDNA. We are interested in understanding the relationship between DNA structure and AuNPs colloidal stability. Recently, we reported a novel assembly pathway for the one-dimensional DNA polymer chain preparation, and the target DNA was determined by the naked eye based on the different effects of the hairpin DNA probes and one-dimensional DNA polymer chains on the stability of AuNPs solution^13^. However, up to now, little is known about how hairpin DNA structure affects the stability of AuNPs solution. We speculated that the improved understanding of the behavior of this entity will facilitate the development of applications in colorimetric biosensor.

## Results and Discussion

### Unusual Sequence Length-Dependent AuNPs Aggregation

**Figure 1:**
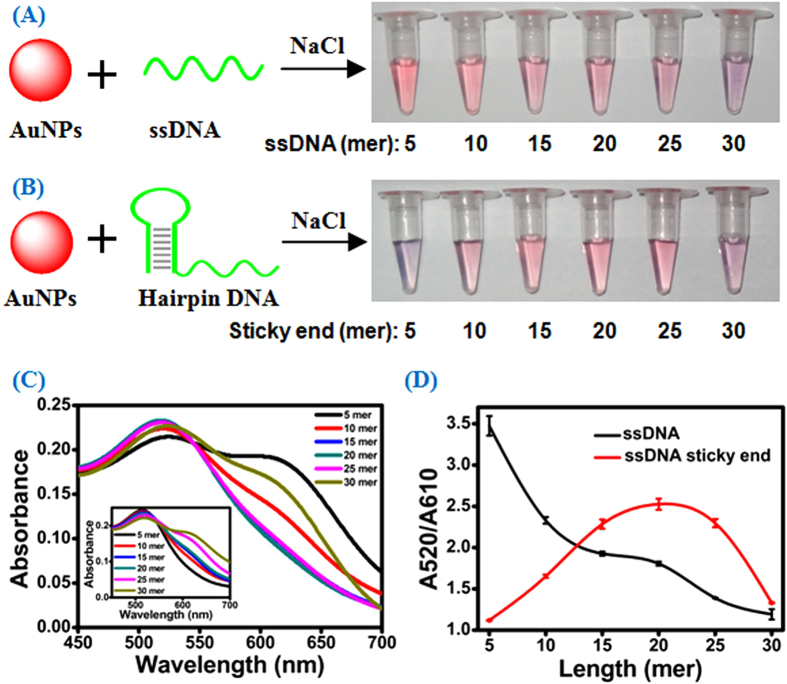
(**A**) Photographs of AuNPs solutions in the presence of ssDNAs with different lengths. (**B**) Photographs of AuNPs solutions in the presence of hairpin DNAs with different lengths of ssDNA sticky ends. (**C**) The absorption spectra of AuNPs in the presence of hairpin DNAs with different lengths of ssDNA sticky ends after NaCl addition. Inset: The absorption spectra of AuNPs in the presence of ssDNAs with different lengths after NaCl addition. (**D**) Absorbance ratio (A520/A610) showing colorimetric responses of AuNPs solution with ssDNAs and hairpin DNAs.

**Figure 2:**
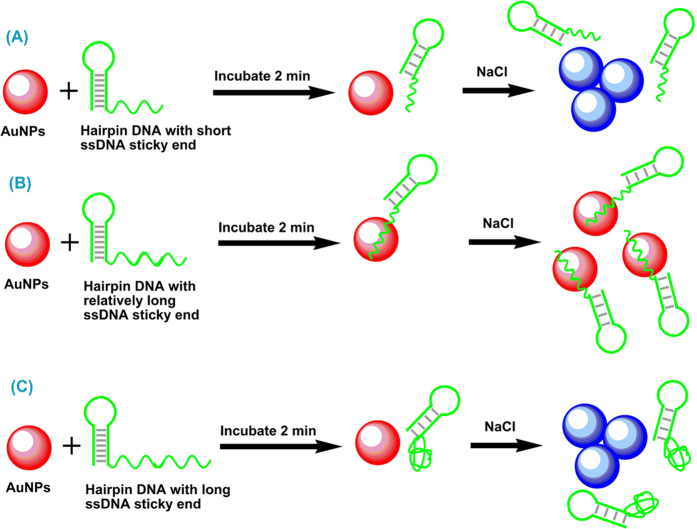
The adsorption behavior of hairpin DNA with different lengths of ssDNA sticky ends on AuNPs. (**A**) It is difficult for the hairpin DNA with short sticky ends to adsorb onto AuNPs and can not prevent salt-induced AuNPs aggregation, the color of solution becomes blue. (**B**) Hairpin DNA with relatively long sticky ends can adsorb onto AuNPs and the color of the solutions sequentially changed from blue to red with the increase of sticky end length. (**C**) Long ssDNA sticky end tends to form coiled configuration and prevent the hairpin DNA to adsorb onto AuNPs, then the color of the solutions gradually varied from red to blue with the increase of sticky end length.

In the present study, several hairpin DNAs with 18 base-pair stem and different lengths of ssDNA sticky ends, varying from 5- to 30-mer, were examined using unmodified AuNPs (DNA sequences were listed in Table S1 in the supporting information). ssDNAs with the same sequence to the sticky ends of hairpin DNAs were used as control. In order to achieve the best colorimetric performance, the experimental conditions including the concentrations of hairpin probes and AuNPs, and the additional amount of NaCl were optimized (Figure S1). The 2 min incubation time was directly used according to the literature reported^21^. After 2 min incubation of AuNPs with hairpin DNA or ssDNA, NaCl was added immediately. For the ssDNA, it exhibited length-dependent AuNPs aggregation and the color of the AuNPs solutions gradually changed from red to blue with the increase of ssDNA length (Fig. 1A), which is similar to the literature reported^21^. For the sticky end with the length longer than 20-mer, the color of the solutions also changed progressively from red to blue with the increase of sticky end length (Fig. 1B). Meanwhile, the maximum absorbance at 520 nm in UV-vis absorption spectra gradually decreased and the broad absorption around 610 nm gradually increased (Fig. 1C). The color variation and spectra data are similar to those of ssDNAs. However, for the ssDNA sticky end with the length shorter than 20-mer, a surprising blue-to-red color variation of the solutions was observed with the increase of sticky end length (Fig. 1B). Accordingly, the broad absorption around 610 nm gradually disappeared and the absorbance at 520 nm gradually increased (Fig. 1C). The color variation and spectra data are contrary to those of ssDNAs, indicating that the sticky end exhibits different adsorption behavior on AuNPs compared to ssDNA. In order to evaluate the aggregation state of AuNPs in more detail, the ratio of absorbance at 520 nm and 610 nm (A520/A610) was used to assess the degree of AuNPs aggregation (Fig. 1D). The ratio is also associated with the color of the AuNPs solution, with a low ratio corresponding to a blue solution and a high ratio corresponding to a red one. It is obvious that ssDNA sticky end exhibits an unusual length-dependent AuNPs aggregation compared to ssDNA. We speculated that this unique phenomenon might be ascribed to the effect of the stem of hairpin DNA. The stem with double-helix structure is rigid and always presents the negatively charged phosphate backbone, which leads to strong repulsion between stem and negatively charged AuNPs. Since the short ssDNA sticky end (e.g., 5-mer) with less bases exposed, van der Waals attraction between short sticky end and AuNPs is weak compares to the repulsion between stem and AuNPs, thus it is hard for the short sticky ends to adsorb onto AuNPs (Fig. 2A). As a result, bare AuNPs can not prevent salt-induced aggregation and the color of solution becomes blue (Fig. 1B). For the relatively long sticky ends (e.g., 10-, 15- and 20-mer), van der Waals attraction between sticky end and AuNPs gradually becomes stronger as the sticky end length becomes larger. Therefore, sticky end can adsorb onto AuNPs gradually (Fig. 2B) and the color of the solutions sequentially changed from blue to red with the increase of sticky end length (Fig. 1B). However, if the ssDNA sticky end is too long (e.g., 25- and 30-mer), it tends to form coiled configuration and the negatively charged phosphate backbone is most exposed to the aqueous solution^20^. Therefore, the repulsion between coiled sticky end and AuNPs plus the repulsion between stem and AuNPs will prevent sticky end to adsorb onto AuNPs (Fig. 2C), then the color of the solutions gradually varied from red to blue with the increase of sticky end length (Fig. 1B). Further evidence for this hypothesis can be obtained by examining the interaction between AuNPs and hairpin DNAs with 10 and 14 bp stem. Since short stem has less negative charges, the repulsion between stem and AuNPs is relatively weak, making the sticky end easy to adsorb onto AuNPs and preventing salt-induced aggregation. As expected, the color of AuNPs solutions becomes more and more red in accordance with the decrease of stem length (Figure S2). Based on the above results, different color of the AuNPs solution can be easily obtained by altering the length of stem and sticky end of the hairpin DNA. This is a potential and useful mechanism for sensing application.

### Application in DNA detection

**Figure 3:**
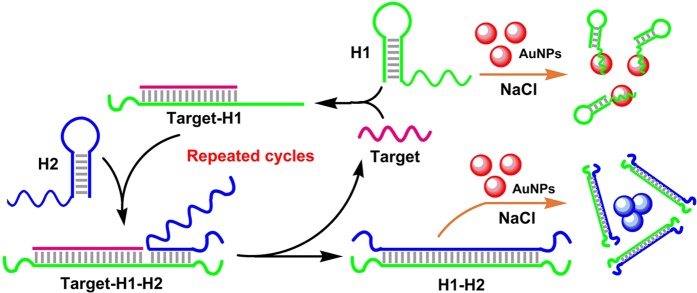
Principle for DNA detection based on the unusual length-dependent AuNPs aggregation and catalytic hairpin assembly.

In this work, a novel AuNPs-based and signal amplified colorimetric DNA assay was proposed based on this unusual length-dependent AuNPs aggregation and catalytic hairpin assembly (CHA)^24^,^25^. The detection principle is illustrated in Fig. 3. Two hairpin probes, H1 and H2, both with 18 bp stem and 20-mer sticky end, were designed to partially hybridize to each other (DNA sequences were listed in Table S2 in the supporting information). However, the spontaneous hybridization between H1 and H2 was kinetically hindered by caging complementary sequences in the stems of the hairpins^26^,^27^. Thus they can maintain the stem-loop structure in the absence of the target and prevent salt-induced aggregation according to the results shown above. In the presence of the target DNA, it hybridizes with and opens the hairpin structure of H1 by the principle of the toehold-mediated DNA strand displacement. The newly exposed sticky end of H1 nucleates at the sticky end of H2 and triggers second strand-displacement reaction to release the target. Thus, the released target is able to act as a catalyst to trigger another reaction cycle for the formation of H1-H2 complexes. The recycle of the target results in amplification of the detection signals. Since the lengths of the stem and the sticky ends of the H1-H2 complex are 50 bp and 9- and 13-mer, respectively, according to the unusual length-dependent AuNPs aggregation mechanism we proposed above, this H1-H2 complex with a such long stem and short sticky end cannot prevent salt-induced AuNPs aggregation.

**Figure 4:**
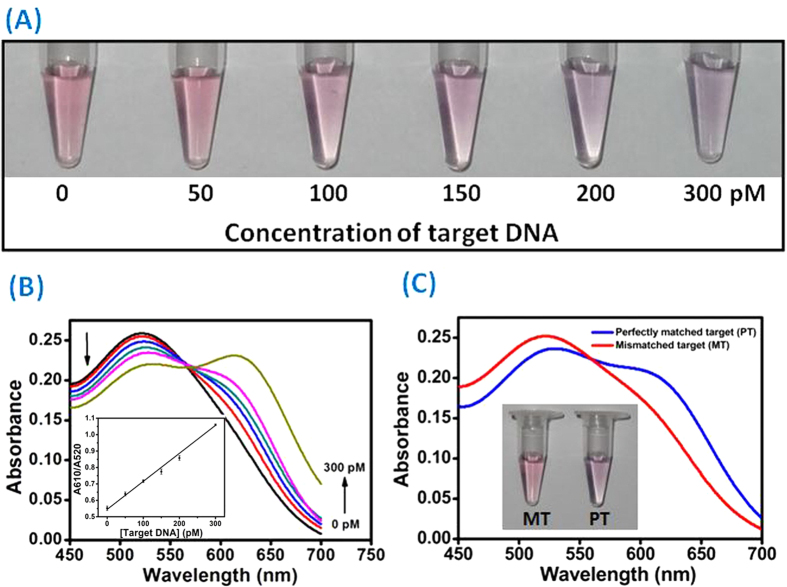
(**A**) Photograph showing colorimetric responses of the assay in the presence of various concentrations of target DNA. (**B**) Absorption spectra for AuNPs-based colorimetric detection of target DNA. Inset: Plot of target DNA concentration vs absorbance ratio (A610/A520) for the target DNA assay. (**C**) Absorption spectra of AuNPs in the presence of perfectly matched target DNA (PT) and single-base mismatched target DNA (MT). Inset: Photographs of AuNPs solutions in the presence of PT and MT.

In order to achieve the best assay performance, the assay conditions including the reaction time, the concentrations of hairpin probes and AuNPs, and the additional amount of NaCl were optimized (Figure S3), and the obtained optimum reaction conditions were carefully chosen in the subsequent experiments. Figure 4A demonstrates the concentration profile of the target DNA-induced color change. With the increase of target concentration, the color of the AuNPs solution gradually turned to dark purple, implying an increase in the aggregation of AuNPs. We could observe the color change for a target DNA concentration as low as 100 pM by naked eyes. The UV-vis data presented in Fig. 4B shows that along with the increasing of the target DNA concentration, the absorbance at 520 nm decreased and the broad absorption around 610 nm gradually increased. Accordingly, the absorbance peak ratio at 610 nm and 520 nm was employed to quantitatively scale the target DNA concentration. A linear dependence of the A610/A520 ratio on the DNA concentration in the range of 50 pM to 300 pM was found (Fig. 4B), and a detection limit of 11.3 pM (S/N = 3) was achieved. These results are comparable with the reported AuNPs-based colorimetric DNA assay (Table S3). To validate the sequence-specificity of our detection system, we performed a control experiment using a DNA with a single base mismatch with the target. The result showed that this system is capable of discriminating as low as 200 pM of perfectly matched DNA and single-base mismatched DNA by naked eyes (Fig. 4C). The high sequence specificity of the current strategy is attributed to the relatively long stem of the hairpin probes, which can make the hairpin structure thermodynamically stable, and it is unfavorable for the hybridization between mismatched target DNA and the hairpin probe^26^.

## Conclusions

In conclusion, we have demonstrated that the ssDNA sticky end of hairpin DNA presents a different adsorption behavior on AuNPs compared to ssDNA, and exhibited an unusual sequence length-dependent AuNPs aggregation after addition of salt. We use this unique phenomenon to develop an enzyme-free, signal amplified, highly sensitive and selective colorimetric DNA assay based on the CHA amplification, picomolar sensitivity and specificity for DNA detection by naked eyes was achieved. Another hairpin-based nonenzymatic nucleic acid circuit^28^ or hybridization chain reaction (HCR)^29^,^30^ also can be used for this novel colorimetric sensing strategy. In addition, this strategy might have the inherent advantage for RNA detection because hairpin loop is the most common element of RNA secondary structure. It also provides a novel and general platform for the detection of protein, metal ions, small molecule, and enzyme activity, especially for the restriction enzymes (e.g., EcoRI, BamHI and PstI) that can generate sticky ends. Thus, this unusual sequence length-dependent AuNPs aggregation of the ssDNA sticky end we found here introduces a new direction for the AuNPs-based colorimetric assays.

## Experimental

### Materials and Reagents

Tris-HCl, sodium hydroxide, sodium chloride, magnesium chloride hexahydrate, and Tris-EDTA buffer solution (100×) were purchased from Sigma-Aldrich (Shanghai) Trading Co. Ltd. Hydrogen tetrachloroaurate(III) (HAuCl_4_) was purchased from Shanghai Chemical Reagent Research Institute Co. Ltd. All DNA oligonucleotides were synthesized by Sangon Biotechnology (Shanghai) Co. Ltd. and purified by HPLC. The DNA sequences are listed in Table S1 and Table S2. Other chemicals were used as received without further purification. AuNPs of ~13 nm were synthesized by the citrate reduction of HAuCl_4_ as previously reported^31^. The deionized water was purified using a Millipore filtration system and used in all experiments.

### Apparatus

DNA concentration was measured by Qubit^®^ 2.0 Fluorometer (Thermo Fisher Scientific Inc.). UV-vis absorption spectra were obtained using a UV-1100 spectrophotometer (Techcomp (China) Ltd.). Photographs were taken using a Sony DSC-WX150 digital camera.

### The Adsorption Behaviors of ssDNA and Hairpin DNA on AuNPs

In a typical experiment, ssDNAs and hairpin DNAs were diluted to 2 *μ*M with 50 mM Tris-HCl (pH 8.0) buffer containing 5 mM MgCl_2_, respectively. These DNA solutions were then heated to 95 °C for 5 min, and then allowed to cool to room temperature (25 °C) for at least 3 h before use. Subsequently, 5 *μ*L of each DNA solution was added to an AuNP solution (2.7 nM) of 100 *μ*L. After incubation for 2 min, 10 *μ*L of NaCl (300 mM) was introduced to the mixed solution, followed by either visual observation or UV/Vis characterization.

### AuNPs-based Colorimetric DNA Assay

In a typical target DNA assay, H1 and H2 were heated to 95 °C for 5 min and then allowed to cool to room temperature for 3 h before use. Target DNA samples were mixed with H1 (100 nM) and H2 (100 nM) in 50 mM Tris-HCl (pH 8.0) buffer solution containing 5 mM MgCl_2_, and then incubated for 20 min at room temperature. Subsequently, 5 *μ*L of the reaction solution was added to the 100 *μ*L of AuNP solution (2.7 nM) and incubated for 2 min, followed by addition of 10 *μ*L of NaCl (90 mM) for colorimetric detection.

## Additional Information

**How to cite this article**: He, H. *et al.* Unusual sequence length-dependent gold nanoparticles aggregation of the ssDNA sticky end and its application for enzyme-free and signal amplified colorimetric DNA detection. *Sci. Rep.* **6**, 30878; doi: 10.1038/srep30878 (2016).

## Supplementary Material

Supplementary Information
