# Supplementary material for: Unusual sequence length-dependent gold nanoparticles aggregation of the ssDNA sticky end and its application for enzyme-free and signal amplified colorimetric DNA detection
Source: Sci Rep. 2016 Aug 1;6:30878. doi: 10.1038/srep30878 (PMC4967886; doi:10.1038/srep30878)
Supplement: Supplementary Information [file srep30878-s1.doc]

**Supporting Information**

Unusual sequence length-dependent gold nanoparticles aggregation of the ssDNA sticky end and its application for enzyme-free and signal amplified colorimetric DNA detection

Hongfei He^1^, Jianyuan Dai^1*^, Zhijuan Duan^1^, Baozhan Zheng^1^, Yan Meng^2^, Yong Guo^1,*^, Dan Xiao^1,2*^

^1^College of Chemistry, Sichuan University, 29 Wangjiang Road, Chengdu 610064, People’s Republic of China

^2^College of Chemical Engineering, Sichuan University, No.24 South Section 1, Yihuan Road, Chengdu 610065, People’s Republic of China

**Table S1 DNA sequences of ssDNA and hairpin DNA with different lengths of stem (10, 14 and 18 bp) and ssDNA sticky ends (5, 10, 15, 20, 25 and 30 mer) *^a^***

| DNA name | DNA sequence (5’ to 3’) |
| --- | --- |
| ssDNA-1 | GTAGT |
| ssDNA-2 | GACAAGTAGT |
| ssDNA-3 | TTCTCAACAAGTAGT |
| ssDNA-4 | AGCAATTCTCAACAAGTAGT |
| ssDNA-5 | AGTCCAGCAATTCTCAACAAGTAGT |
| ssDNA-6 | CAGATAGTCCAGCAATTCTCAACAAGTAGT |
| Hairpin-10-5 | GTAGTGATTGAGCGTTCACTACTTCAACTCGACGCTCAATC |
| Hairpin-10-10 | GACAAGTAGTGATTGAGCGTTCACTACTTCAACTCGACGCTCAATC |
| Hairpin-10-15 | TTCTCAACAAGTAGTGATTGAGCGTTCACTACTTCAACTCGACGCTCAATC |
| Hairpin-10-20 | AGCAATTCTCAACAAGTAGTGATTGAGCGTTCACTACTTCAACTCGACGCTCAATC |
| Hairpin-10-25 | AGTCCAGCAATTCTCAACAAGTAGTGATTGAGCGTTCACTACTTCAACTCGACGCTCAATC |
| Hairpin-10-30 | CAGATAGTCCAGCAATTCTCAACAAGTAGTGATTGAGCGTTCACTACTTCAACTCGACGCTCAATC |
| Hairpin-14-5 | GTAGTGATTGAGCGTGATGTCACTACTTCAACTCGCATCACGCTCAATC |
| Hairpin-14-10 | GACAAGTAGTGATTGAGCGTGATGTCACTACTTCAACTCGCATCACGCTCAATC |
| Hairpin-14-15 | TTCTCAACAAGTAGTGATTGAGCGTGATGTCACTACTTCAACTCGCATCACGCTCAATC |
| Hairpin-14-20 | AGCAATTCTCAACAAGTAGTGATTGAGCGTGATGTCACTACTTCAACTCGCATCACGCTCAATC |
| Hairpin-14-25 | AGTCCAGCAATTCTCAACAAGTAGTGATTGAGCGTGATGTCACTACTTCAACTCGCATCACGCTCAATC |
| Hairpin-14-30 | CAGATAGTCCAGCAATTCTCAACAAGTAGTGATTGAGCGTGATGTCACTACTTCAACTCGCATCACGCTCAATC |
| Hairpin-18-5 | GTAGTGATTGAGCGTGATGAATGTCACTACTTCAACTCGCATTCATCACGCTCAATC |
| Hairpin-18-10 | GACAAGTAGTGATTGAGCGTGATGAATGTCACTACTTCAACTCGCATTCATCACGCTCAATC |
| Hairpin-18-15 | TTCTCAACAAGTAGTGATTGAGCGTGATGAATGTCACTACTTCAACTCGCATTCATCACGCTCAATC |
| Hairpin-18-20 | AGCAATTCTCAACAAGTAGTGATTGAGCGTGATGAATGTCACTACTTCAACTCGCATTCATCACGCTCAATC |
| Hairpin-18-25 | AGTCCAGCAATTCTCAACAAGTAGTGATTGAGCGTGATGAATGTCACTACTTCAACTCGCATTCATCACGCTCAATC |
| Hairpin-18-30 | CAGATAGTCCAGCAATTCTCAACAAGTAGTGATTGAGCGTGATGAATGTCACTACTTCAACTCGCATTCATCACGCTCAATC |

*^a^* The design of the hairpin probes was adapted from the literature^[1]^.

***Table S2 DNA sequences used in AuNPs colorimetric DNA detection***a

| DNA name | DNA sequence (5’ to 3’) |
| --- | --- |
| H-1 | AGCAATTCTCAACAAGTAGTGATTGAGCGTGATGAATGTCACTACTTCAACTCGCATTCATCACGCTCAATC |
| H-2 | AGCAATTCTTAACTGATGAATGCGAGTTGAAGTAGTGACATTCATCACGCTCAATCACTACTTCAACTCGCA |
| Target | GACATTCATCACGCTCAATCACTACTT |
| Mismatched target | GACATTCATCACGTTCAATCACTACTT |

*^a^* The design of the hairpin probes was adapted from the literature^[1]^. The part of the sequence marked in red denotes the mismatched base.

**Table S3: Comparison of the present method with other reported AuNPs-based colorimetric DNA detection methods.**

| AuNPs | Reaction time  (min) | Dynamic range | Detection limit  (pM) | References |
| --- | --- | --- | --- | --- |
| Unmodified | 60 | 50 pM-500 pM | 50 | 2 |
| Modified | 15 | 25 pM-10000 pM | 11.2 | 3 |
| Unmodified | 180 | 50 pM-700 pM | 25 | 4 |
| Unmodified | 20 | 50 pM-300 pM | 11.3 | This work |

**
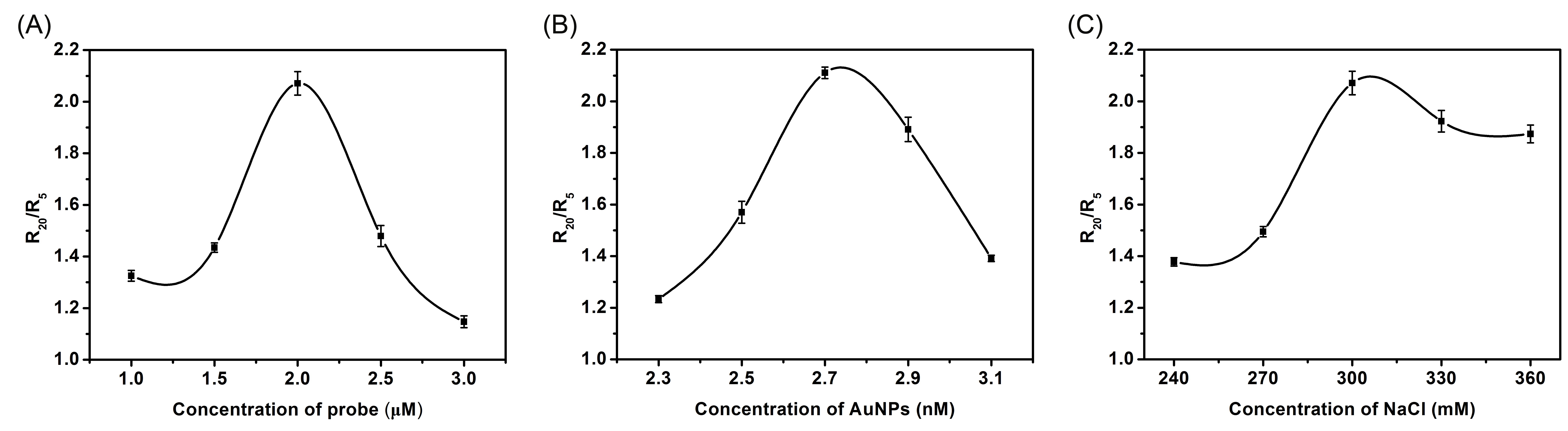
**

Figure S1. Condition optimization for hairpin DNAs with 18 base-pair stem: Concentrations of (A) hairpin probes, (B) AuNPs and (C) NaCl. R_20_/R_5_: The ratio of A520/A610 (hairpin DNA with 20-mer ssDNA sticky end) versus A520/A610 (hairpin DNA with 5-mer ssDNA sticky end).


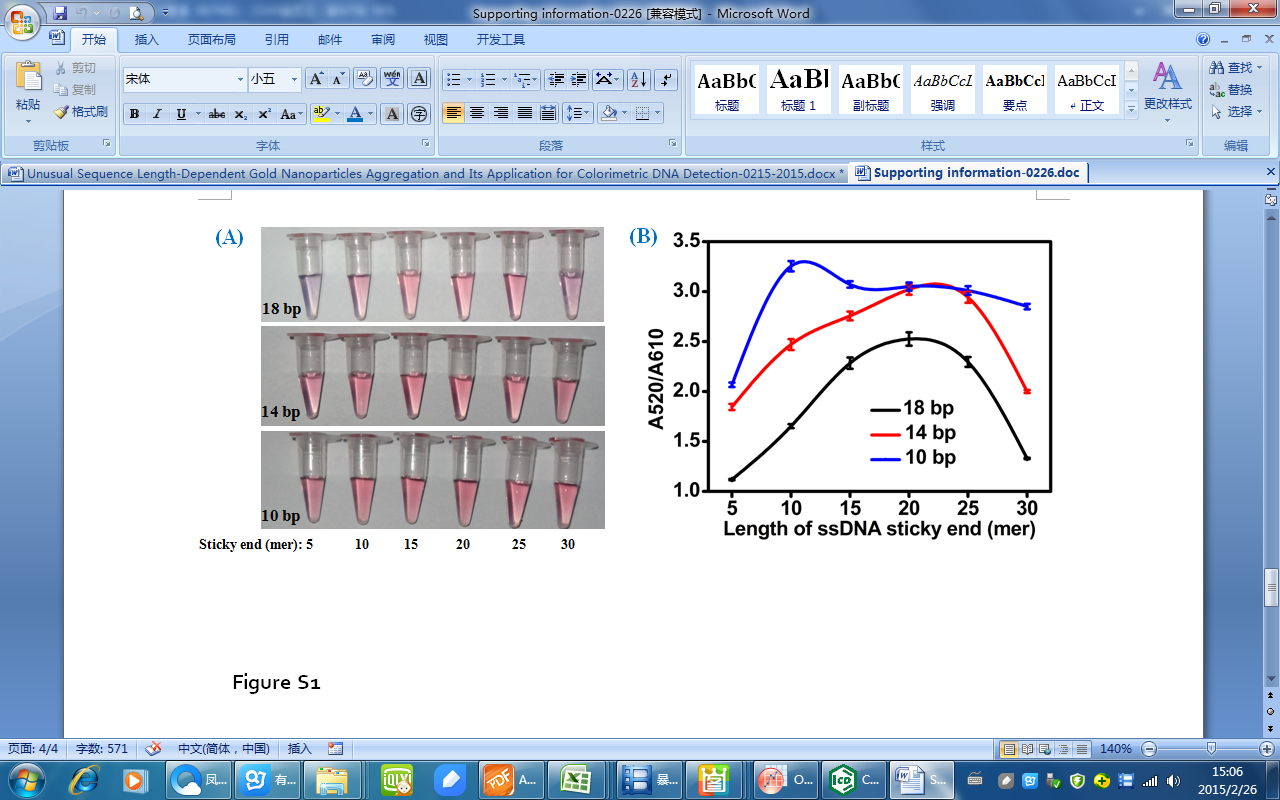


Figure S2. (A) Photographs of AuNPs solutions in the presence of different hairpin DNAs. (B) Absorbance ratio (A520/A610) showing colorimetric responses of AuNPs solution with different hairpin DNAs.


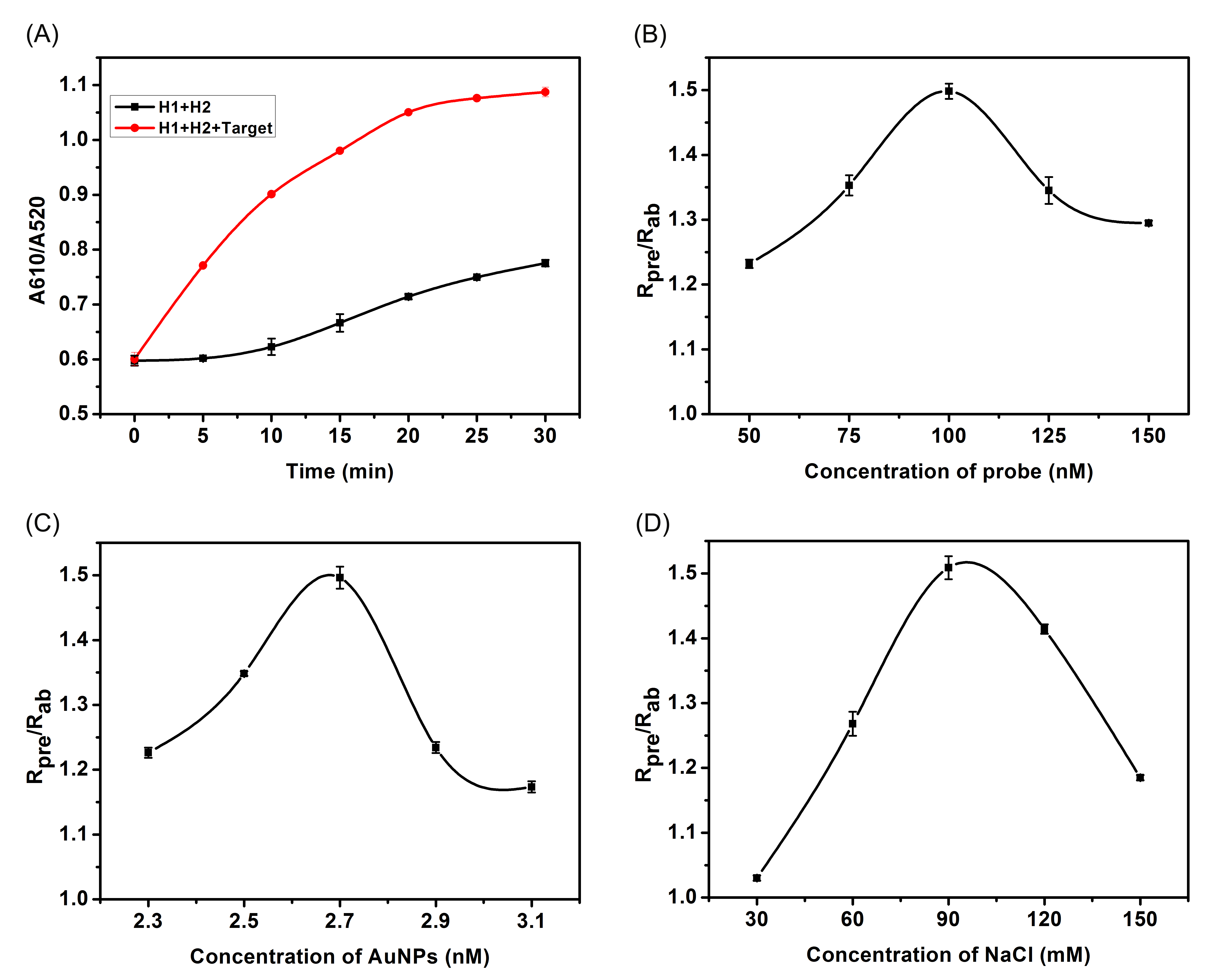


Figure S3. Condition optimization for nucleic acids detection: (A) reaction time, Concentrations of (B) hairpin probes, (C) AuNPs and (D) NaCl. R_pre_/R_ab_: The ratio of A610/A520 (in the presence of 300 pM target DNA) versus A610/A520 (in the absence of target DNA).

**References**

[1] Huang, J. H., Su, X. F. & Li, Z. G. Enzyme-free and amplified fluorescence DNA detection using bimolecular beacons. *Anal. Chem.* **84**, 5939–5943 (2012).

[2] Liu, P. et al. Enzyme-free colorimetric detection of DNA by using gold nanoparticles and hybridization chain reaction amplification. *Anal. Chem.* **85**, 7689−7695 (2013).

[3] Ma, C. P. et al. Three-dimensional DNA nanostructures for colorimetric assay of nucleic acids. *J. Mater. Chem. B*, **3**, 2853−2857 (2015).

[4] Dai, J. Y. et al. Target-triggered autonomous assembly of DNA polymer chains and its application in colorimetric nucleic acid detection. *J. Mater. Chem. B.* **4**, 3191−3194 (2016).
